# Intracellular MIZU-KUSSEI1 movement and hydrotropism in *Arabidopsis* require F-actin organization

**DOI:** 10.1093/plphys/kiaf495

**Published:** 2025-10-08

**Authors:** Kotaro Akita, Yutaka Miyazawa

**Affiliations:** Graduate School of Science and Engineering, Yamagata University, 1-4-12 Kojirakawa-machi, Yamagata-shi, Yamagata 990-8560, Japan; Faculty of Science, Yamagata University, 1-4-12 Kojirakawa-machi, Yamagata-shi, Yamagata 990-8560, Japan

## Abstract

Tropism is directed growth toward or away from stimuli such as light, gravity, and moisture gradient, by which plants can adapt to their surroundings. Hydrotropism is a response to a moisture gradient across the soil, which enables plants to grow their roots toward wet soil and thus avoid drought conditions. *MIZU-KUSSEI 1* (*MIZ1*) is a pivotal gene for root hydrotropism, and its function inside cortical cells at the transition zone is indispensable for hydrotropic bending. However, how MIZ1 is regulated in the cortical cells remains unclear. Here, we found that treatment with the actin depolymerizing drug latrunculin B (Lat B) reduces hydrotropic bending of the *Arabidopsis* (*Arabidopsis thaliana*) wild-type and a *MIZ1* overexpression line. Moreover, lines with knocked down actin depolymerizing factors showed enhanced hydrotropic root bending, partly due to an increase in *MIZ1* expression. We further explored intracellular MIZ1 dynamics using a GFP-fused MIZ1 (MIZ1-GFP) expressing line and found that MIZ1-GFP moves in the cytosol. Lat B treatment diminished MIZ1-GFP movement, indicating the movement of MIZ1-GFP is actin-dependent. These results indicate that actin filaments are required for proper hydrotropic root bending, probably by controlling MIZ1 expression and localization.

## Teaser

Pharmacological and genetic analyses reveal that the intracellular movement of MIZ1 (a protein necessary for root hydrotropism) and root hydrotropism require intact F-actin organization.

## Introduction

Land plants must respond appropriately to their surroundings in order to survive. Among various environmental responses in plants, tropism, a response of an organ toward or away from a directional stimulus, is one of the most well-known responses. For instance, in response to the gravity vector, plants shoots display negative gravitropism, by which the shoots grow upward, and thus shoot organs can be irradiated by the sunlight, helping them efficiently perform photosynthesis. Also, shoot positive phototropism, a shoot bending toward the light source, helps maximizing photosynthesis. In case of roots, where uptake of water and nutrients occur, efficient acquisition of water is critical for plants’ survival especially when water is limited. Under water-limited condition, positive gravitropism and negative phototropism play roles in orienting roots deeper in soil, and thus roots are able to avoid drying. More directly, plant roots respond to moisture gradients across the soil and display hydrotropism, by which the roots bend toward a moistened area. In maize and *Arabidopsis*, root hydrotropism has been proved to enhance biomass production and survival rate under water-limited condition, relatively less is understood when compared with phototropism or gravitropism (Iwata et al. 2013; Eapen et al. 2017).

Previous molecular genetic studies using *Arabidopsis* have revealed many factors involved in root hydrotropism (reviewed in Miyazawa and Takahashi 2020). Among them, *MIZU-KUSSEI 1* (*MIZ1*) and *MIZ2* are considered as central genes, for their malfunctions result in complete loss of hydrotropism (Kobayashi et al. 2007; Miyazawa et al. 2009). *MIZ1* encodes an uncharacterized protein, while *MIZ2* encodes a guanine-nucleotide-exchange factor for ARF GTPase. Although the biochemical role of MIZ1 is unknown, MIZ1 contains a plant specific domain of unknown function (DUF) 617, which is termed MIZ1-domain. Because overexpression of *MIZ1* confers enhancement of hydrotropic response as well as water stress tolerance in *Arabidopsis*, it is suggested that MIZ1 is thought to be a positive regulator of hydrotropism (Miyazawa et al. 2012). Recent analyses showed that MIZ1-domain containing protein also functions in hydrotropism in tomato (Wexler et al. 2024a). Similarly, it is suggested that an MIZ1-domain containing protein in rice plays a role in drought and osmotic stress responses (Kaur et al. 2020). The molecular mechanism underlying hydrotropism differs among the plant species (reviewed in Miyazawa and Takahashi 2020); nevertheless, these results suggest that MIZ1 and MIZ1-domain containing proteins play indispensable roles in water stress-related response, irrespective of plant species.

As above, the indispensable role of MIZ1 on root hydrotropism is well established; however information about biochemical properties of MIZ1 is still limited. Confocal microscopic observation of transgenic plants expressing green fluorescent protein (GFP)-fused MIZ1 showed that MIZ1 was observed to be localized in cytosol, and some of which were observed to be colocalized with ER marker (Yamazaki et al. 2012). Further biochemical experiments confirmed that MIZ1-GFP appeared in both microsomal and soluble fractions, and our sucrose density gradient fractionation combined with protease protection assay showed that MIZ1-GFP precipitated with microsome was associated with cytoplasmic face of ER membrane (Yamazaki et al. 2012). Indeed, Shkolnik et al. (2018) showed that an ER-localized calcium pump, ECA1, generated Ca^2+^ signal upon hydrostimulation at columella root cap cells and was negatively regulated by direct interaction with MIZ1. In addition, recent results indicated that this regulation depends on phosphorylation of MIZ1 (Ju et al. 2024). On the other hand, MIZ1 is not only expressed at columella cells but also at lateral root cap and cortical and epidermal cells around the elongation zone (Yamazaki et al. 2012). Together with the fact that MIZ1 expressed only at cortical cells is responsible for root hydrotropism (Dietrich et al. 2017), it is suggested that cortical cells around the elongation zone have a dual function during a hydrotropic response, both sensing a moisture gradient and subsequent differential growth (Dietrich et al. 2017). However, how MIZ1 acts inside the cortical cells has never been investigated.

Recently, we found that MIZ1 is required not only in the early phase but also in the latter phase of hydrotropism for fine-tuning the root curvature (Akita and Miyazawa 2023). As hydrotropic root bending seems to be dependent on differential growth of cortical cells at the elongation zone, it is likely that regulatory function of MIZ1 at this phase is related to cell elongation. Several lines of evidence have shown that dynamics of actin filaments profoundly affect cell elongation (García-González and van Gelderen 2021). Actin filament is maintained by the balance between polymerization of globular actin (G-actin) subunits from their barbed (plus) end and depolymerization of the subunits from their pointed (minus) end. Organization of actin filaments is mediated by actin-binding proteins that nucleate, destabilize, and bundle actin filaments. The key regulators of actin nucleation and subsequently polymerization are the ACTIN RELATED PROTEIN 2/3 (ARP2/3) complex and its activator, suppressor of cAMP receptor (SCAR)/WASP family verprolin homologous (WAVE) complex, and FORMINs (Blanchoin and Staiger 2010; Yanagisawa et al. 2013). In animals and yeast, the ARP2/3 complex consists of 7 subunits: ARP2, ARP3, and ACTIN RELATED PROTEIN C 1-5 (ARPC1-5) (Machesky and Gould 1999). FORMINs are characterized by the presence of a formin homology-2 (FH2) domain (Blanchoin and Staiger 2010). All of the putative subunits of the *ARP2/3* complex, 4 functional SCAR isoforms, 5 subunits of WAVE complex, and 21 isoforms of FORMIN have been identified in the genome of *Arabidopsis thaliana* (Blanchoin and Staiger 2010; Yanagisawa et al. 2013). Both ARP2/3 complex and FORMINs independently polymerize actin filaments. Among the subunits of the SCAR/WAVE complex, *BRICK1*/*HSPC300* (*BRK1*) specifically functions as a component of the WAVE complex (Dyachok et al. 2008). BRK1 functions with ARP2/3 complex and protects SCAR1 from degradation (Djakovic et al. 2006). Additionally, the reduced root growth phenotype of *brk1* mutant is similar to that of *arp2*, *arp3*, and *scar1234* mutants (Dyachok et al. 2008). Thus, *brk1* is a suitable model for studying the role of SCAR/WAVE complex-mediated actin polymerization. On the other hand, actin depolymerization is mediated by actin depolymerization factors (ADFs)/cofilin. In *Arabidopsis*, 11 ADFs are encoded in the genome (Ruzicka et al. 2007). This expansion of gene number has allowed these genes for functional diversity to environmental stimuli. Moreover, dynamics of actin filaments are modulated by environmental stimuli as well as plant hormones such as auxin and abscisic acid, both of which are involved in hydrotropic responses (Antoni et al. 2013; Scheuring et al. 2016; Dietrich et al. 2017; Akita and Miyazawa 2023). From these, we postulated that dynamics of actin filaments are involved in regulating root hydrotropic bending and investigated it physiologically.

## Results

### Intact actin filaments are necessary for appropriate hydrotropic bending

**Figure 1.**
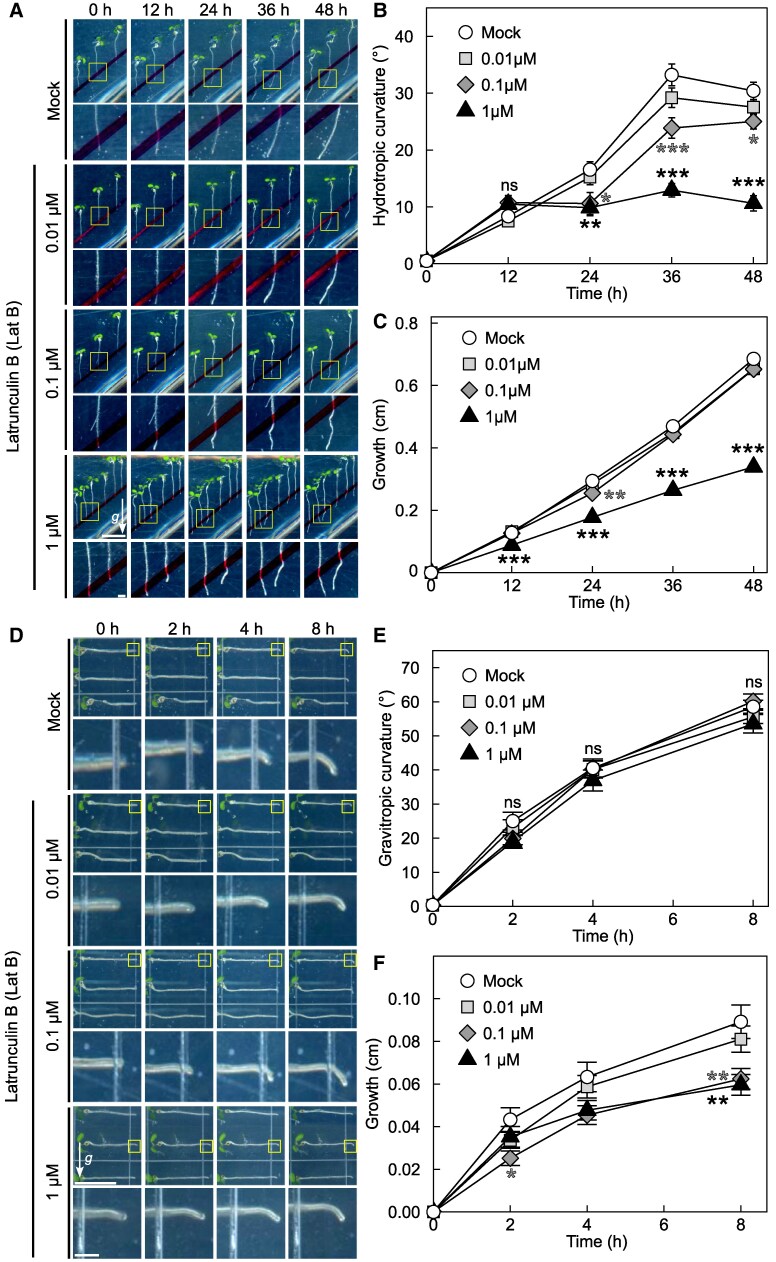
Effect of Lat B treatment on hydrotropism and gravitropism. Wild-type seedlings were pretreated with Lat B at indicated concentrations and subjected to the assays. For control experiments, seedlings were placed on media only containing dimethylsulfoxide, a solvent used to dissolve Lat B. Hydrotropic and gravitropic root curvatures were measured at indicated times. Representative images, their magnified images of root tip (framed with yellow boxes), curvature and growth of hydrotropically responding roots **A** to **C)**, and gravitropically responding roots **D** to **F)** are shown. Arrows (*g*) indicate the direction of gravity and scale bars represent 1 cm (top) and 1 mm (bottom), respectively. Gravity vector and scale bars are applicable for all images. Data in **B**, **C**, **E**, and **F)** are the means ± SE from 59 to 60 roots from 3 independent experiments. Asterisks denote significant difference between mock- and Lat B-treated samples, as determined by Dunnett's test (*P < 0.05; **P < 0.01; ***P < 0.001). “ns”, no significant differences.

The dynamics of actin filaments are regulated by actin polymerization and depolymerization. To explore the role of actin filaments in hydrotropic response, we conducted the dose–response experiment using actin depolymerizing drug, latrunculin B (Lat B) (Fig. 1, A to C). The hydrotropic root bending and growth were reduced in a dose-dependent manner. No significant differences were observed between mock-treated and 0.01 *µ*M Lat B-treated samples. On the other hand, Lat B treatment above 0.1 *µ*M significantly reduced root hydrotropic bending compared with the mock-treatment samples. Root growth was not affected by treatment of Lat B at 0.1 *µ*M, while it was significantly decreased by treatment with 1 *µ*M Lat B when compared with mock-treated samples. In many plant species, including *Arabidopsis*, root hydrotropism interferes with gravitropism (reviewed in Wexler et al. 2024b). Given that gravitropism was also affected by Lat B treatment, whether disruption of actin filament by Lat B directly affected hydrotropism or not remains unclear. Indeed, previous studies had demonstrated that root gravitropism in *Arabidopsis* seedling was enhanced by Lat B treatment (Hou et al. 2004; Zou et al. 2016). To examine this, we monitored gravitropism of Lat B-treated seedling roots. Under our experimental setup, we could not detect significant increase nor decrease in gravitropism of Lat B-treated roots, when compared with mock-treated control, irrespective of the concentration of Lat B (Fig. 1, D and E). Because treatments with Lat B at higher concentrations reduced root growth, we believe that Lat B properly affected to the samples (Fig. 1F). Thus, these results indicate that intact actin filaments are necessary for the regulation of appropriate hydrotropic bending.

### BRICK1/HSPC300 (BRK1), an activator of actin polymerization, is required for root hydrotropic bending

**Figure 2.**
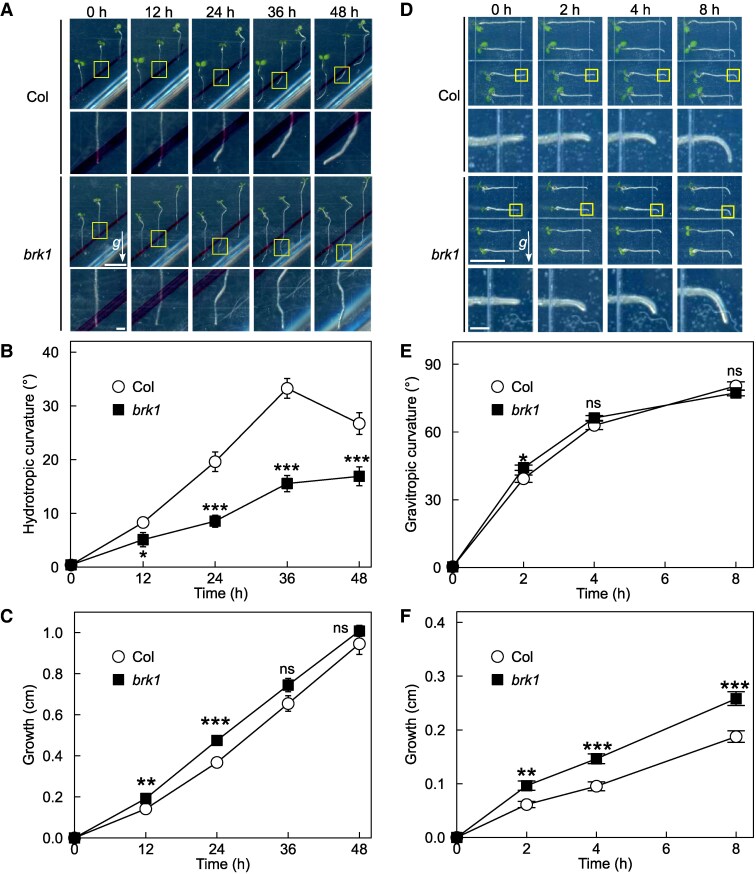
Hydrotropic and gravitropic responses of *brk1* mutant. Seedlings of the wild type and *brk1* were subjected to either hydrotropism or gravitropism assay. Representative images (top), enlarged views of root tips framed with yellow boxes (bottom), curvature and growth of hydrotropically responding roots **A** to **C)** and gravitropically responding roots **D** to **F)** are shown. Arrows (*g*) indicate the direction of gravity, and scale bars represent 1 cm (top) and 1 mm (bottom) and are applicable for all images, respectively. Data in **B**, **C**, **E**, and **F)** represent means ± SE from 55 to 60 roots from 3 independent experiments. Significant differences observed between Col and *brk1* are indicated by asterisks, as determined by Welch's *t*-test (*P < 0.05; **P < 0.01; ***P < 0.001). Nonsignificant differences are denoted as “ns”.

To further confirm that actin filament is involved in root hydrotropism in *Arabidopsis*, using mutants defective in actin itself or its dynamics. In vegetative tissues of *Arabidopsis*, ACTIN2 (ACT2), ACTIN7 (ACT7), and ACTIN8 (ACT8) is expressed. Of these, ACT7 has been proved to play a major role in root growth (Takatsuka et al. 2018). Although mutant alleles of *ACT7* are available, their growth and germination are severely affected by mutation so that they are unsuited for analyzing hydrotropism. Because of this reason, the use of mutants defective in actin polymerization is an alternative way to analyze the involvement of actin dynamics in hydrotropism. In this sense, we decided to use a mutant defective in *BRICK1*/*HSPC300* (*BRK1*). BRK1 is a component of “suppressor of cAMP receptor defects/Wiskott-Aldrich syndrome protein family verprolin homologous protein (SCAR/WAVE)” complex, and it serves as an activator of ACTIN RELATED PROTEIN 2/3 (ARP2/3) complex, a nucleator of F-actin polymerization (Frank et al. 2004; Dyachok et al. 2008). Indeed, loss of function mutant of BRK1 (*brk1*) showed a decrease in overall root growth as well as cortical cell expansion at the elongation zone under illuminated condition (Dyachok et al. 2008, 2011). As shown in Fig. 2, A to C, root hydrotropic bending was significantly diminished in *brk1* mutant, besides its elongation was almost comparable to wild type. Furthermore, root gravitropic response of *brk1* was similar to that of the wild type, although root growth was enhanced in *brk1* when compared with the wild type (Fig. 2, D to F). These observations coincided well with our previous results that Lat B treatment inhibited root hydrotropism.

### Subclass I actin depolymerizing factors (ADFs) attenuate hydrotropic bending

**Figure 3.**
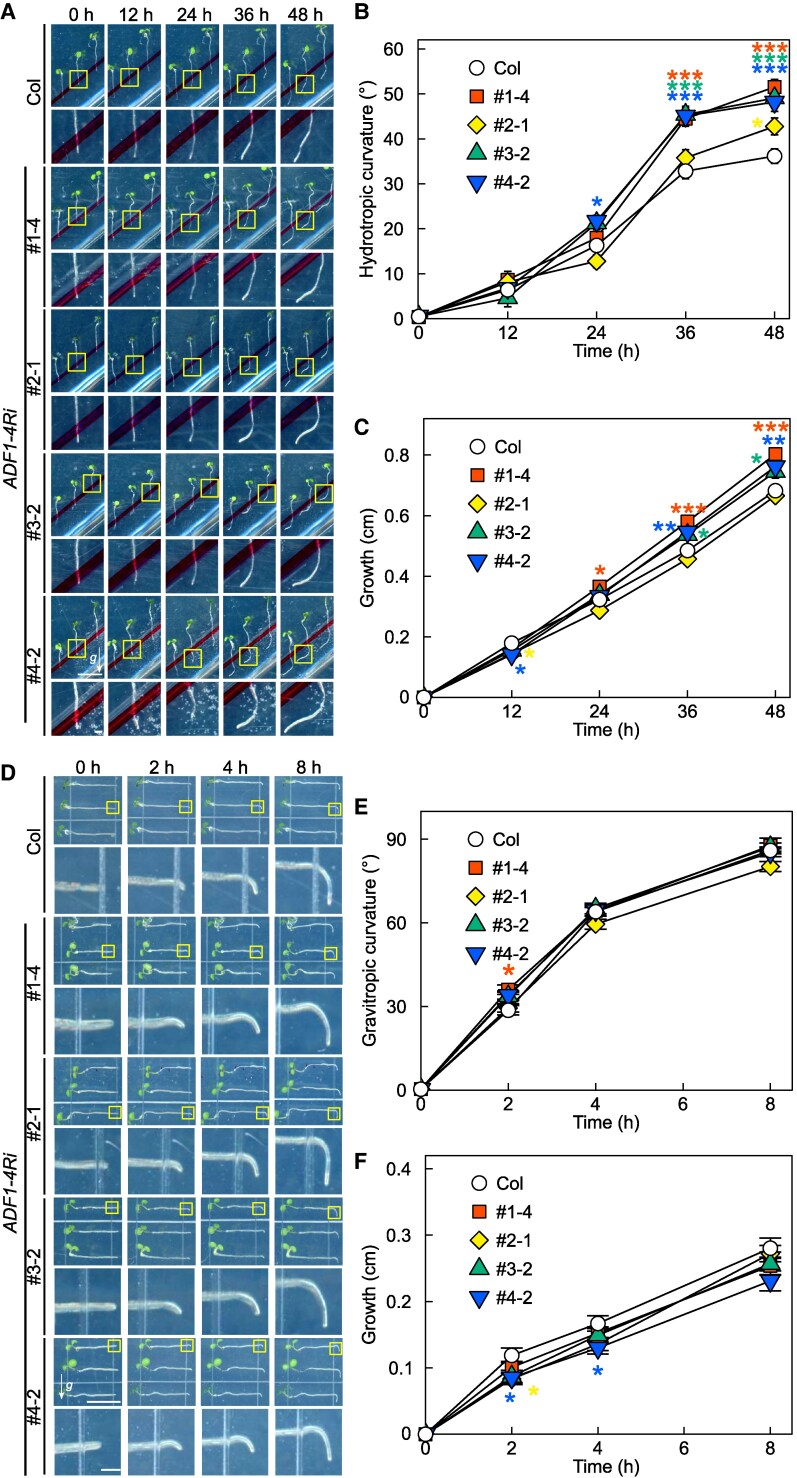
Hydrotropic and gravitropic responses of *ADF1-4* knockdown mutants. Panels **A** and **D)** are the representative images (top) and their enlarged views (bottom) of hydrotropic and gravitropic response of Col and 4 RNA*i* lines of *ADF1-4* (*ADF1-4Ri*). Arrows (*g*) show the direction of gravity, and scale bars represent 1 cm (top) and 1 mm (bottom), and magnified images of root tips framed with yellow boxes are shown, respectively. Direction of gravity and scale bars are applicable for all images. Hydrotropic and gravitropic root curvatures are shown in **B** and **E)**, respectively. Root growth upon hydrostimulation **C)** and gravistimulation **F)** are also presented. Data represent means ± SE from 59 to 60 roots across 3 independent experiments. Asterisks denote significant differences observed between Col and *ADF1-4Ri* lines as determined by Dunnett's test (*P < 0.05; **P < 0.01; ***P < 0.001; ns, *P* > 0.05).

Dynamics of actin filaments are not only regulated by its polymerization but also by its depolymerization. If actin filament organization is necessary for proper root hydrotropism, it is likely that actin depolymerization also plays a role in root hydrotropism. ADF plays a crucial role in regulating actin dynamics. *A. thaliana* has 11 ADFs, and among them, ADF1, ADF2, ADF3, and ADF4 are expressed predominantly in vegetative tissues including seedling root (Ruzicka et al. 2007). Due to the presumed redundancy of their functions, the use of lines in which ADF1-4 are simultaneously knocked down by RNA interference (*ADF1-4Ri*) is one of the choices for investigating the physiological role of these ADFs (Tian et al. 2009; Inada et al. 2016). In this study, we used 4 independent lines of *ADF1-4Ri*, namely, #1-4, #2-1, #3-2, and #4-2. During the late stages of hydrotropism, the root bending and growth in each *ADF1-4Ri* line was significantly enhanced (Fig. 3, A to C). Similar enhancement of root hydrotropic bending was also observed in *ADF1-4Ri* lines exposed to humidity-based hydrotropism assay (Supplementary Figs. S1B, S2). Coinciding to the effect of pharmacological and genetic depolymerization of actin filament, the effect of inhibition of actin depolymerization seemed to be prominent at the latter phase of hydrotropic root bending. On the other hand, the degree of gravitropic bending of *ADF1-4Ri* roots was almost equivalent to that of the wild type, except that a slight increase in gravitropic curvature was observed in line #1-4 at 2 h post gravistimulation (Fig. 3, D and E). Similarly, root growth of *ADF1-4Ri* lines during gravitropic response was almost the same as those of the wild type, besides only little decrease in root growth was observed in lines #3-2 and #4-2 (Fig. 3F).

### MIZ1 function is dependent on dynamics of actin filament

**Figure 4.**
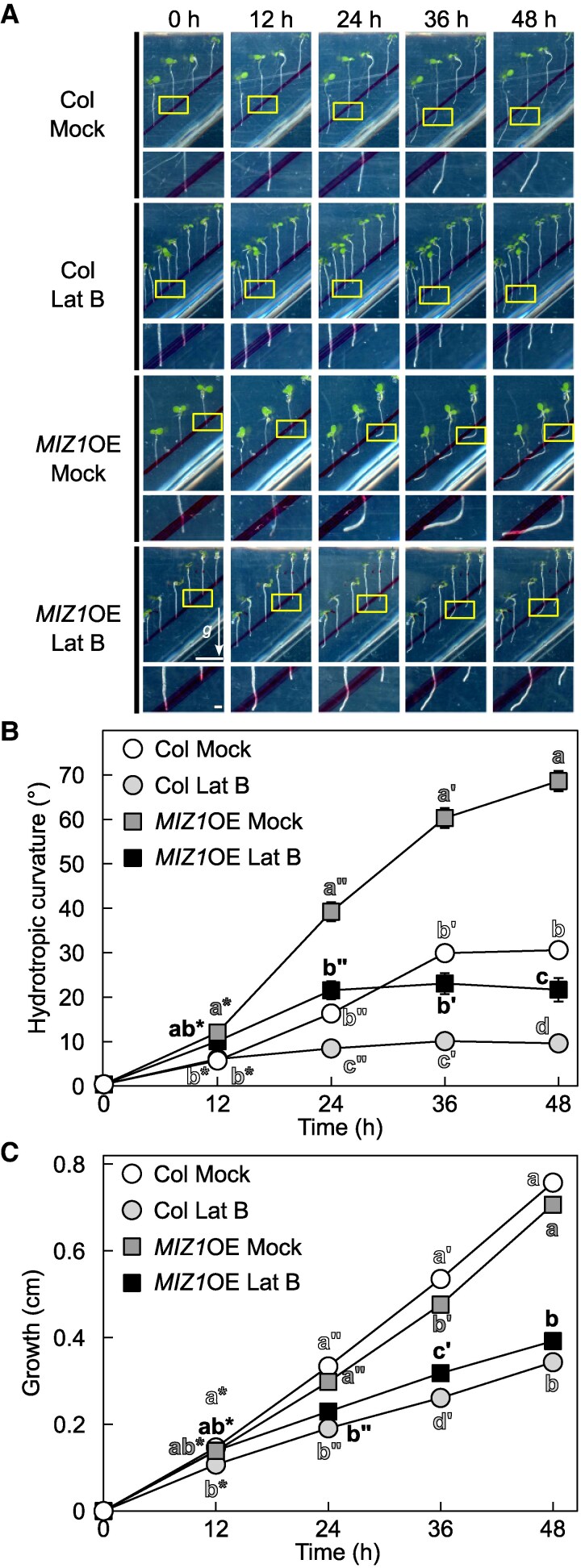
Effect of Lat B treatment on hydrotropic response of *MIZ1*OE. **A)** Representative images (top) with their magnified images (bottom) of control and 1 *µ*M Lat B-treated roots in response to hydrostimulation are shown. Yellow boxes represent the regions used for acquiring magnified images. An arrow (*g*) and scale bars represent gravity vector and 1 cm (top) and 1 mm (bottom) and are applicable for all images, respectively. **B)** Changes in hydrotropic root curvature. **C)** Changes in root growth. Data in **B** and **C)** are the means ± SE from 59 to 60 roots across 3 independent experiments, and the different letters denote significant differences, as determined by Tukey's honestly difference test (*P* < 0.05).

**Figure 5.**
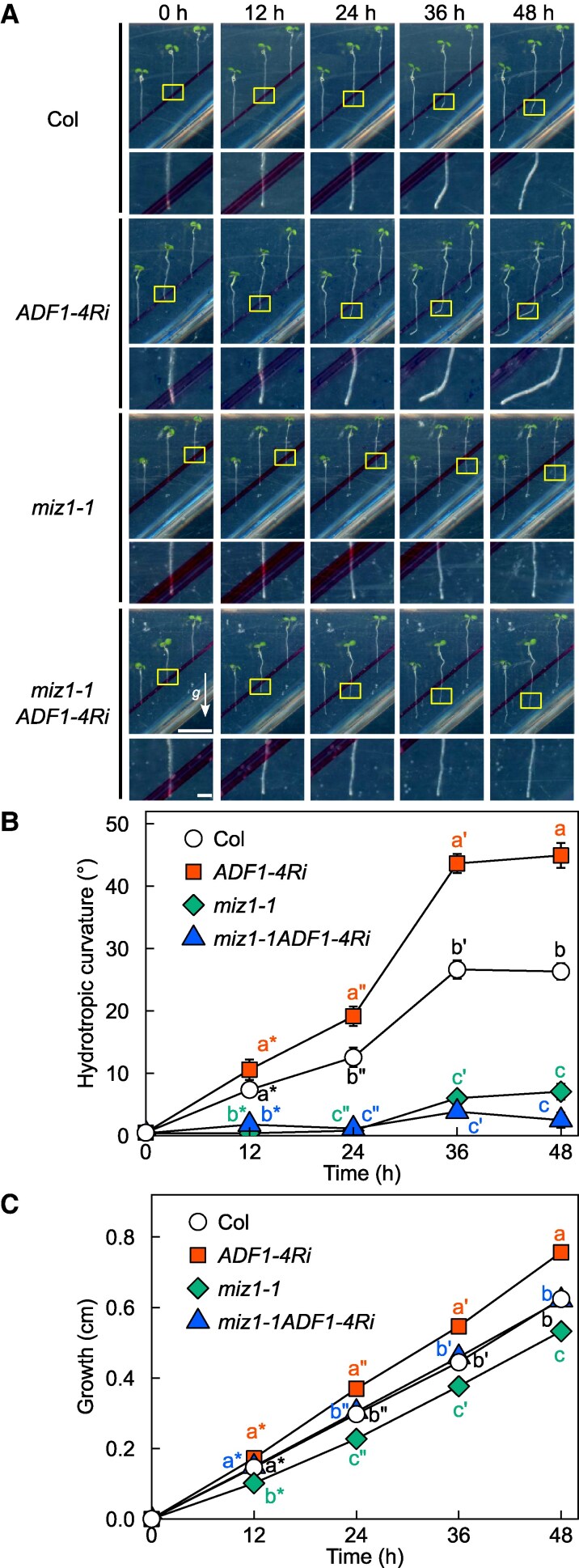
Effect of *miz1-1* mutation on *ADF1-4Ri* lines on hydrotropic response. Representative images (top) with their magnified images (bottom) **A)**, changes in curvature **B)** and growth **C)** of hydrotropically responding roots of Col, *miz1-1*, *ADF1-4Ri*, and *miz1-1ADF1-4Ri* are shown. Yellow boxes represent the regions used for acquiring magnified images. An arrow and scale bars in **A)** represent gravity vector and 1 cm (top) and 1 mm (bottom), respectively. Gravity vector and scale bars are applicable for all images, respectively. Data in **B** and **C)** represent means ± SE from 60 roots across 3 independent experiments. Different letters denote significant differences among each genotype as determined by Tukey's honestly difference test (*P* < 0.05).

As current results suggested a direct involvement of actin dynamics in modulating root hydrotropic bending, especially during its latter stage, it is intriguing to know whether function of actin filament is related to MIZ1, an essential protein for hydrotropism. To understand this, we treated Lat B to both wild type and overexpressor of *MIZ1* (*MIZ1*OE) and investigated the effect of Lat B treatment by comparing them with mock-treated ones (Fig. 4, A to C). Although *MIZ1*OE has been reported to show enhanced hydrotropic response, the effect of MIZ1 overexpression was clearly diminished by Lat B treatment, especially at the latter stage of root hydrotropism. Furthermore, we generated an *ADF1-4Ri* line carrying *miz1-1* mutation (referred to as *miz1-1ADF1-4*Ri, hereafter) and investigated its hydrotropic phenotype (Fig. 5, A to C). In wild-type background, knocking down the subclass I ADFs enhanced root hydrotropic root bending, especially at its latter stage; however such effect was completely nullified by introducing the *miz1-1* allele. These results clearly demonstrated that ADF-regulated actin dynamics functioned in the same pathway where MIZ1 functioned, and MIZ1 function on root hydrotropism relied on dynamics of actin filament.

**Figure 6.**
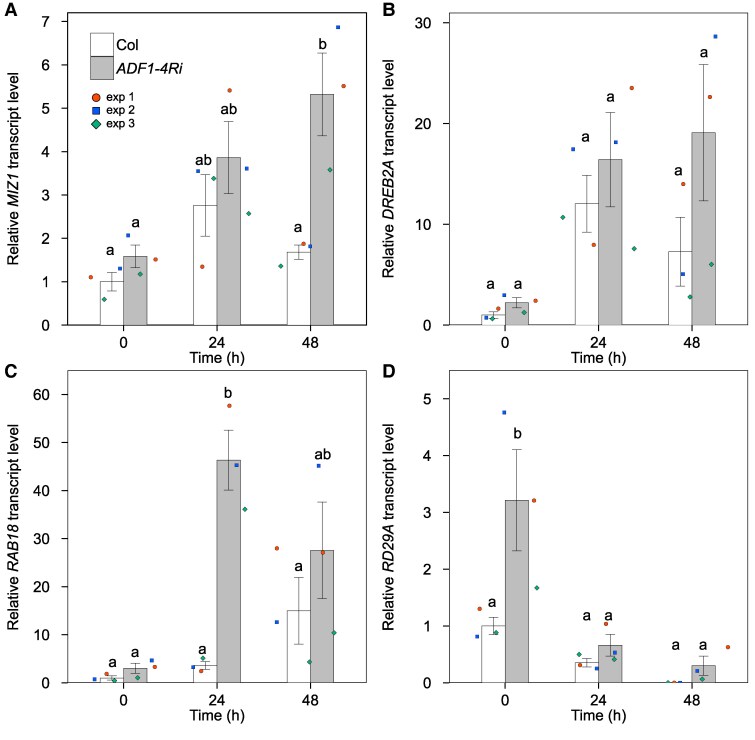
Comparison of transcript levels of *MIZ1* and the osmotic stress-responsive maker genes, *DREB2A*, *RAB18*, and *RD29A* between the wild type and *ADF1-4Ri* during root hydrotropism. Hydrostimulated roots of wild type (Col) and *ADF1-4Ri* #3-2 were sampled at 24 h intervals and subjected to RT-qPCR analyses. Transcript abundance of *MIZ1*  **A)**, *DREB2A*  **B)**, *RAB18*  **C)**, and *RD29A*  **D)** are shown. Abundance of *rRNA* was used for normalization. Accumulation of mRNA is expressed as a relative value, where the accumulation of Col at 0 h is set as 1. Data are the means ± SE (*n* = 3). Datum from each experiment is presented by different colors. Different alphabets represent significant differences as determined by Tukey's honestly difference test (*P* < 0.05).

We next investigated how subclass I ADFs attenuate hydrotropic bending. Recently, Matsumoto et al. (2023) reported that subclass I ADFs regulate nuclear organization and gene expression. Following to this, we monitored the changes in *MIZ1* mRNA levels of *ADF1-4Ri* during hydrotropism and compared with those of wild type, because the transcript level of *MIZ1* is closely related to the degree of hydrotropic root bending (Fig. 6A). During the early stage of hydrotropism, *MIZ1* mRNA levels in *ADF1-4Ri* were slightly, but not significantly, more abundant than those in the wild type. On the other hand, at 48 h after hydrostimulation, abundance of *MIZ1* mRNA in *ADF1-4Ri* was significantly higher than that of the wild type. It should be noted that in *ADF1-4Ri*, *MIZ1* expression was still increasing even at 48 h after hydrostimulation at which *MIZ1* gene expression in the wild type was diminished to attenuate the root hydrotropic bending. This might be the reason why *ADF1-4Ri* did not cease root bending at the latter stage of hydrotropism.

In the split-agar-based hydrotropism assay, sorbitol is diffused according to the time passed from the addition of 1% agar containing sorbitol. Because of these characteristics of this experimental setup, the 1% agar nearby the roots might be surrounded by the diffused sorbitol, especially at the prolonged exposure to hydrostimulation. Thus, it would be informative to investigate whether the elevated mRNA level of *MIZ1* in *ADF1-4Ri* reflects a directional water gradient or a nondirectional osmotic stress. We first assessed the mRNA accumulation levels of the osmotic stress marker genes, *DREB2A*, *RAB18*, and *RD29A* during the split-agar-based hydrotropism assay. Overall, the transcript levels of these osmotic stress marker genes were tended to highly maintained in *ADF1-4Ri* compared with that of the wild type (Fig. 6, B to D). Notably, the expression level of *RD29A* was significantly elevated even before hydrostimulation in *ADF1-4Ri* (Fig. 6D). These data suggest that *ADF1-4Ri* responds more sensitive than the wild type in terms of osmotic stress-responsive gene expressions. Next, to test whether exposure to nondirectional osmotic stress itself can upregulate *MIZ1* gene expression or not, we investigated the root growth and the transcript levels of *MIZ1* in both the wild type and *ADF1-4Ri* under the condition where sorbitol was uniformly applied in 1% agar. In split-agar-based experiment, sorbitol concentration will be approximately 400 mm when sorbitol is evenly diffused throughout the agar. From this reason, we set maximum sorbitol concentration at 400 mm. When roots were grown on 1% agar containing 400 mm sorbitol, the roots ceased their growth irrespective of the genotypes (Supplementary Fig. S3, A and B). Rather, the growth of hydrostimulated roots resembled to that grown under 1% agar containing 200 mm sorbitol. Thus, the roots under hydrostimulated condition seem to be exposed to moderate osmotic stress. Although transcript levels of *MIZ1* did not significantly differ among the conditions, the MIZ1 transcript level was maintained high in *ADF1-4Ri* treated with 200 mm sorbitol for 48 h (Supplementary Fig. S3C). Regarding the osmotic stress marker genes, no significant differences were detected when compared at the same time points (Supplementary Fig. S3, D and E). These results suggest that the higher *MIZ1* accumulation level observed in *ADF1-4Ri* under prolonged hydrostimulation was associated with osmotic stress responses.

### MIZ1 movement is dependent on actin filaments

**Figure 7.**
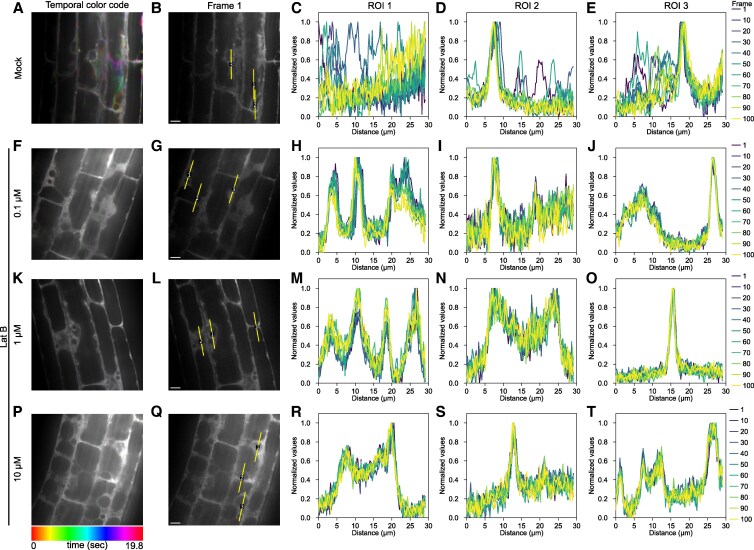
Effect of Lat B treatment on subcellular dynamics of MIZ1. Seedlings were pretreated for 30 min in a liquid 1/2 × MS (pH 5.8) medium containing Lat B and subjected to observation. For control, seedlings were incubated for 30 min in a liquid medium containing dimethylsulfoxide. Time-course images of GFP-fused MIZ1 (MIZ1-GFP) were captured at 200 ms intervals under confocal microscope. Typical images of control **A** and **B)** roots and roots treated with 0.1 *µ*M **F** and **G)**, 1 *µ*M **K** and **L)**, and 10 *µ*M **P** and **Q)** Lat B are shown. Scale bars represent 10 *µ*m and are applicable to all images. The “Spectrum” look-up table was applied to assign colors to the different 100 frames. **B**, **G**, **L**, and **Q)** show the first frame images under each condition. Yellow lines represent the region of interests (ROIs), which were drawn from the bottom to the top. Fluorescent intensity profiles along the yellow lines on **B**, **G**, **L**, and **Q)** are shown in **C** to **E)**, **H** to **J)**, **M** to **O)**, and **R** to **T)**, respectively.

In addition to the regulation of MIZ1 expression, actin filament might have roles in subcellular dynamics of MIZ1 protein to attenuate hydrotropic root bending. To verify this possibility, we captured MIZ1 dynamic as time-lapse images under confocal microscope and analyzed whether it is dependent on actin filament organization. Real-time observation of MIZ1-GFP revealed that MIZ1 dynamically moved between the peripheral and interior region inside the root cortical cells, probably passing through the trans vacuolar regions of the cells (Fig. 7, A to E; Supplementary video S1). This movement of MIZ1 was inhibited by treating Lat B in a dose-dependent manner (Fig. 7, F to T; Supplementary video S2 to S4). In root cells where Lat B was treated at the concentration of 10 *µ*M, MIZ1 movement was rarely observed. We further investigated whether movement of MIZ1 requires MIZ2/GNOM function. GNOM is an ARF-GEF required for vesicle trafficking, and it is well-known that actin filament has an important role in vesicle trafficking; however, there is no evidence that *miz2* mutation affects membrane trafficking. On the other hand, intimate relationships between MIZ1 and MIZ2 have been reported. For example, the effect of *MIZ1* overexpression, such as reduced lateral root formation, enhanced hydrotropic response, and reduced IAA contents, was suppressed in *miz2* background (Miyazawa et al. 2012). Moreover, both MIZ1 and MIZ2 functions in root cortical cells are necessary for complete root hydrotropism (Dietrich et al. 2017; Pang et al. 2023). Thus, it is possible that MIZ2 mediates MIZ1 and actin by controlling vesicle trafficking. Unexpectedly, monitoring of MIZ1 movement on *miz2* background revealed that MIZ1 movement was also observed in *miz2* background (Supplementary Fig. S4; Supplementary video S5). These results revealed that MIZ1 movement requires intact actin filaments, but is not dependent on MIZ2 function.

## Discussion

Our pharmacological and genetic inhibition of actin polymerization/depolymerization resulted in altered hydrotropic bending (Figs. 1, A and B, 2, A and B, 3, A and B) suggesting that the intact actin filaments are necessary for normal hydrotropic bending. In addition, we identified that the SCAR/WAVE complex was involved in this process. *brk1* exhibited reduced hydrotropic bending but its phenotype was milder when compared with 1 *µ*M Lat B-treated wild-type (*cf.* Figs 1, A and B, 2, A and B). This difference of hydrotropic phenotype might be explained by the degree of actin filament destruction between Lat B treatment and *brk1*. Previous studies demonstrated that the treatment of 1 *µ*M Lat B almost completely disrupted the actin filaments, while actin filaments observed in *brk1* were not entirely disrupted but were partially depleted at cell corners on root tips (Dyachok et al. 2008; van der Honing et al. 2011). Besides ARP2/3 complex, FORMINs also take place in actin polymerization (Cheung and Wu 2004). Therefore, knocking-out both regulatory units might phenocopy Lat B-treated seedlings. Because 21 *FORMINs* are encoded in *Arabidopsis* genome, it is hard to analyze the knockout phenotype of each FORMIN at this state. Further study is needed to determine whether the FORMINs are also involved in root hydrotropism. On the other hand, we also observed that hydrotropic root bending was enhanced in *ADF1-4Ri* whose organization of actin filaments was reported to be normal under ordinary condition (Inada et al. 2016). Because actin filament density in *ADF1-4Ri* were significantly altered after infection of *Golovinomyces orontii* (Inada et al. 2016), it is possible that ADFs also alter the organization of actin filaments upon other environmental stimuli, such as hydrostimulation and osmotic stress. Additionally, we examined the expression patterns of the osmotic stress-responsive genes under hydrostimulation as well as under osmotic stress conditions (Fig. 6, Supplementary Fig. S3, C to E). Overall, the expression levels of these makers tended to be elevated in *ADF1-4Ri* compared with the wild type. Matsumoto et al. (2023) reported that many genes were either upregulated or downregulated in *adf4* and *ADF1-4Ri* in mature leaves. Their GO enrichment analyses revealed that genes responsive to stresses, including biotic stress, were either upregulated or downregulated in *adf4* mutant as well as *ADF1-4Ri*. Because the effect of RNA*i* is systemic, it is possible that osmotic stress-related gene expression is also altered in the roots of *ADF1-4Ri*. Although the roles of ADFs in shoots have been extensively investigated, their roles in roots remain poorly understood. Although future research is needed to clarify how ADFs and actin filament organization regulate gene expression in root tissues, however, our current results may open up the interesting possibility that ADFs also regulate gene expression in roots.

The plant cytoskeleton is comprised of actin filaments and microtubules, both of which uniquely and interactively respond to abiotic stress. In *Arabidopsis*, disruption of microtubules and mutations in *KATANIN1*, a microtubule-severing protein, result in reduced root hydrotropic bending and growth (Miao et al. 2022). Similarly, mutations in *BRK1*, an activator of actin polymerization, lead to reduced hydrotropic root bending while maintaining normal root growth. Although both actin filaments and microtubules play positive roles in hydrotropism, they may have distinct functions at different stages of the response. Comparing our current results and the hydrotropism of KATANIN-deficient mutant, it is likely that actin filaments contribute primarily to asymmetric growth, while microtubules are involved in both asymmetric growth and subsequent general growth during hydrotropism. Disrupting the cytoskeleton often causes side effects, such as abnormalities in cell wall construction and morphogenesis. Malfunctions in components of the ARP2/3 and SCAR/WAVE complexes, including *BRK1*, result in distorted trichome phenotypes due to defects in actin filament organization and cell wall assembly (Mathur et al. 2003; Deeks et al. 2004; El-Assal et al. 2004; Zhang et al. 2005; Djakovic et al. 2006; Dyachok et al. 2008). Notably, defects in the cell wall have been associated with increased hydrotropic bending (Chang et al. 2024). Given the apparent inconsistency between the hydrotropic phenotypes observed in cell wall-disrupted mutants and actin filament-disrupted mutants, our results strongly support the idea that actin filaments have a distinct role in hydrotropism, apart from that of the cell wall.

The alteration of hydrotropic root bending was prominent at the latter stage of hydrotropism in both inhibition of actin polymerization and depolymerization, suggesting that actin filaments dynamics are involved in modulation of root growth direction mainly at the latter stage. During the latter stage of hydrotropism, roots cease directional growth toward higher water potential, and then roots bend and grow toward gravitational direction. Generally, soil moisture is influenced by precipitation, soil properties, and seasonal fluctuations that occur in both shallow and deep soil (Gavrilescu 2021; Hoover et al. 2021). In addition, the concurrent effect of drought is stronger in shallow soil than deep soil (Hoover et al. 2021). Thus, it is reasonable for a plant's survival to grow roots deeper in soil when the water is available. This means that the mechanism of not only initiation but also cessation of root hydrotropism is important for plants’ survival. However, the molecular mechanism of the latter is not extensively studied so far. Several lines of evidence have shown that auxin and actin are functionally linked (reviewed in Zhu and Geisler 2015). For example, it has been shown that auxin simultaneously increases actin filament density and decreases actin bundling, which inhibits cell growth (Arieti and Staiger 2020). In addition to our previous results that auxin synthesis, transport, and response negatively regulate root hydrotropic bending, our current results indicated the importance of actin filament organization on root hydrotropism (Fig. 3, A and B; Akita and Miyazawa 2023). Considering the intimate relationship between auxin response and regulation of actin dynamics, it is possible that, in the latter stage of hydrotropism, auxin response alters actin dynamics to inhibit cell growth and thus contribute to fine-tuning of root bending. Although this hypothesis remains to be clarified in the future, our current discovery sheds light on the importance of actin dynamics on plant cell biology.

In many plant species, such as pea and cucumber, it has been shown that root gravitropism strongly interferes with root hydrotropism (Miyazawa and Takahashi 2020). In such cases, root gravitropism dominates hydrotropism on Earth, and root hydrotropism can only be observed under clinorotating conditions (Morohashi et al. 2017; Nakajima et al. 2017). In case of *Arabidopsis*, root gravitropism also interferes with hydrotropism, for root hydrotropism is enhanced when roots are hydrostimulated under clinorotating condition, irrespective of experimental setups (Kobayashi et al. 2007; Miyazawa et al. 2012). On the other hand, *Arabidopsis* has been reported to have 2 mechanisms that minimize the effect of gravitropism against hydrotropism. Using humidity-based assay, Takahashi et al. (2003) reported that hydrostimulation induced amyloplast degradation at columella cells and thus reduced root gravitropism. Such suppression of gravitropism is likely to be in case when roots are hydrostimulated in split-agar-based assay, since amyloplast degradation was also observed when roots were exposed to osmotic stress (Takahashi et al. 2003). In addition to amyloplast degradation at columella cells, hydrostimulation reduces gravitropism by altering auxin dynamics. Using split-agar-based assay, we previously demonstrated that MIZ1 fine-tunes hydrotropic bending upstream of auxin action (Akita and Miyazawa 2023). More recently, Zhang et al. (2025) showed that MIZ1 inhibits gravitropism to promote hydrotropism by dynamically adjusting PIN polarity in response to water stress also using split-agar-based assay. Importantly, amyloplast degradation by osmotic stress as well as alteration of gravitropic auxin redistribution occurred within 2 or 3 h (Takahashi et al. 2003; Zhang et al. 2025). Thus, at least in *Arabidopsis*, gravisensing through amyloplast degradation and changes in auxin distribution related to gravitropism are rapidly diminished under hydrostimulated conditions. Considering these facts, it is likely that the effect of gravitropic response is minimized under hydrostimulated condition, irrespective of experimental systems. Although it has been shown that *MIZ1* mRNA accumulates immediately under both drought and osmotic stress conditions, *MIZ1* expression is diminished when light-grown seeding is transferred to dark condition (Miyazawa et al. 2012; Moriwaki et al. 2012). Thus, in the split-agar-based assay, it is not surprising that fluctuation of *MIZ1* expression in the wild type was little throughout the assay, because the roots faced mild osmotic stress in the dark (Fig. 6A; Supplementary Fig. S3). On the contrary, *MIZ1* expression in the *ADF1-4Ri* line at the latter stage was significantly higher than that of the wild type. Considering that a certain number of stress-related gene expressions are enhanced in *ADF1-4Ri* line, our current results suggest that not only intracellular movements of MIZ1 but also its gene expression is affected by actin dynamics.

Although MIZ1 had been identified to regulate hydrotropism in the cortical cells (Dietrich et al. 2017), the characteristics of its behavior in these cells remained unclear. In this study, we found that MIZ1 moved within cortical cells in an actin filament-dependent manner (Fig. 7; Supplementary video S1 to S4). Several lines of evidence showed that cytoplasmic streaming is controlled by actin filament. Indeed, actin filament depolymerization leads to reduced cytoplasmic streaming and ER streaming in *Arabidopsis* (Ueda et al. 2010). Given that MIZ1 localizes both at the cytosol and at the cytosolic surface of ER, its movement might be driven by cytosolic or ER streaming. Cytoplasmic streaming likely influences cell expansion by facilitating the diffusion of nutrients, cell wall precursors, and plant hormones throughout the cell (Tominaga et al. 2013). In *Chara australis*, it has been shown that high osmolarity decreases cytosolic streaming (Tominaga and Tazawa 1981). So far, the relationship between cytoplasmic streaming and hydrotropism is not known. Currently, we do not have an experimental system that enables us to observe cytoplasmic streaming under hydrostimulated condition. However, it would be valuable to investigate it in the near future. We believe that differential physiological events must occur between the convex and concave sides of cortical cells upon hydrostimulation where the dynamics of actin filaments as well as MIZ1 movement take place. The development of an experimental system that enables continuous observation under hydrostimulated conditions may reveal distinct MIZ1 dynamics between the concave and convex sides of cortical cells in the future.

## Materials and methods

### Plant materials

*A. thaliana* ecotype Columbia and its mutants were used in this study. *miz1-1* and overexpression line of *MIZ1* driven by 35S promoter (*MIZ1*OE#7) was previously described (Kobayashi et al. 2007; Miyazawa et al. 2012). Four independent lines of *ADF1-4Ri* (#1-4, #2-1, #3-2, and #4-2) described in Tian et al. (2009) were kindly provided by Prof. Noriko Inada (Osaka Metropolitan University, Japan). *brk1* (CS86554, Dyachok et al. (2008)) was distributed from *Arabidopsis* Biological Resource Center. *miz1-1ADF1-4Ri* and *miz2MIZ1-GFP* were generated by crossing.

### Growth conditions

Seeds were surface sterilized in a solution containing 10% (v/v) regular bleach and 0.45% (v/v) Tween 20 for 10 min. Seeds were rinsed 3 times with sterile water, and then they were planted on a half-strength Murashige and Skoog (MS) medium (pH 5.8) containing 0.4% (w/v) sucrose, which is solidified with 0.3% (w/v) gellan gum. The seeds were incubated in the dark at 4 ℃ for 2 d and were then incubated at 23 ℃ with continuous light. Light conditions are described in Akita and Miyazawa (2023).

### DNA isolation and genotyping

Rosette leaves of 2-wk-old seedlings were harvested and homogenized in an extraction buffer described by Akita and Miyazawa (2023). The lysate was then mixed with an equivalent volume of 2-propanol and centrifuged at 12,000 × *g* for 20 min at 23 ℃. After centrifugation, precipitated DNA was washed with 70% (v/v) ethanol and then resuspended in 200 *µ*L of 10 mm Tris–HCl buffer (pH 8.0). PCR-based genotyping was performed as described in Akita and Miyazawa (2022). Primers used for genotyping are shown on Supplementary Table S1. Restriction endonucleases were purchased from New England Biolabs (Ipswich, MA, U.S.A.).

### Lat B treatment

Lat B was purchased from Wako Pure Chemicals Industries (Osaka, Japan) and Merck (Darmstadt, Germany). It was dissolved in dimethylsulfoxide (DMSO; Nacalai Tesque, Kyoto, Japan). For the control, an equivalent volume of DMSO was added to the medium. Pretreatments were performed by aligning the seedlings, with their roots being straight, on media containing Lat B at designated concentrations for 90 min.

### Hydrotropism and gravitropism assay

Split-agar-based and humidity-based hydrotropism assays were conducted following the protocol described by Kaneyasu et al. (2007). Procedures for both experiments were illustrated in Supplementary Fig. S1, A and B. Results described in Figs. 1, A to C, 2, A to C, 3, A to C, and 4 to 6 were performed using split-agar-based assay, while results described in Supplementary Fig. S2 were done by humidity-based assay. Gravitropism assays were performed on 1% (w/v) agar plates infused with half-strength MS medium supplemented with 0.4% (w/v) sucrose. The plates were vertically positioned then rotated by 90° for gravistimulation. All assays were conducted at 23 ℃ in the dark. The root curvature and length were measured using the Fiji software with SmartRoot plugin (Lobet et al. 2011).

### Osmotic stress treatment

Four-day-old seedlings were transferred onto 1% (w/v) agar plates containing varying concentrations of sorbitol (0, 200, and 400 mm, Nacalai Tesque). To monitor the root responses under similar condition to split-agar-based hydrotropism assay, these agar plates contained neither 1/2 × MS salts nor sucrose. The seedlings were grown vertically at 23 ˚C in the dark condition.

### Gene expression analysis

Total RNA was extracted from seedlings roots using Sepasol RNA I Super G reagent (Nacalai Tesque), according to the manufacturer's instructions. Complementary DNA was synthesized by ReverTra Ace qPCR RT Master Mix Kit with gDNA Remover (Toyobo, Osaka, Japan) using 100 ng of total RNA. RT-qPCR was performed using SsoAdvanced Universal SYBR Green Supermix (Bio-Rad, Hercules, CA, U.S.A.) on the CFX Connect Real-time PCR System (Bio-Rad Laboratories). Gene expressions among samples were normalized with the amounts of rRNA in corresponding samples. Primers for RT-qPCR analyses are listed on Supplementary Table S2.

### Confocal microscopy and image analysis

Seedling roots were mounted on coverslips with half-strength MS liquid medium supplemented with 0.4% (w/v) sucrose. All samples were observed at room temperature. Images were taken using CSU-X1 confocal scanner unit (Yokogawa, Tokyo, Japan) attached to a microscopy (IX71; Olympus, Tokyo, Japan). The GFP was excited by a 488 nm laser. To generate the stacked image from time-lapse images, different colors were assigned for each frame, and the all frames were merged in a single image using Fiji's “Temporal-Color code” function. A time-lapse image was compressed for JPEG format at 5 fps using Fiji, which were used for generating an avi format movie.

For the fluorescent intensity profile analysis shown in Fig. 7 and Supplementary Fig. S4, 100 time-lapse images were acquired in each series. Each image was captured at intervals of 200 ms. The region of interest was set using Fiji's straight-line tool, and the fluorescent intensity of each frame was measured using Fiji's “Plot Profile” function. The acquired intensity data were normalized by the min–max normalization method, where the maximum intensity was set to 1.0.

### Statistical analysis

All the statistical analyses were conducted using the R software (version 4.2.1; https://www.r-project.org/) in RStudio. ANOVA was applied using the “aov” function. For post hoc tests, the “TukeyHSD” or “glht” function were used for Tukey's honestly different test (HSD) or Dunnett's test, respectively. The “aov” and “TukeyHSD” functions are included in the “stats” package, and the “glht” function is contained in the “multicomp” package. “t.test” function in the “stats” package was used for Welch's *t*-test.

## Accession numbers

Sequence data from this article can be found in the GenBank/EMBL data libraries under the following accession numbers: *ADF1* (AT3G46010), *ADF2* (AT3G46000), *ADF3* (AT5G59880), *ADF4* (AT5G59890) *BRK1* (AT2G22640), *DREB2A* (AT5G05410), *MIZ1* (AT2G41660), *MIZ2/GN* (AT1G13980) *RAB18* (AT5G66400), and *RD29A* (AT5G52310).

## Supplementary Material

kiaf495_Supplementary_Data

## Data Availability

The data underlying this article will be shared on reasonable request to the corresponding author.

## Supplementary data

The following materials are available in the online version of this article.

**Supplementary Figure S1.** Hydrotropism experimental systems.

**Supplementary Figure S2.** Hydrotropic responses of *ADF1-4Ri* #2-1 and #3-2 in the humidity-based hydrotropism assay.

**Supplementary Figure S3.** Effect of osmotic stress treatment on Col and *ADF1-4Ri*#3-2.

**Supplementary Figure S4.** Effect of *miz2* mutation on subcellular dynamics of MIZ1.

**Supplementary Table S1.** Primer sets used for genotyping.

**Supplementary Table S2.** Primer sets for RT-qPCR.

**Supplementary Video S1.** MIZ1 movement under control condition corresponding to Fig. 7A.

**Supplementary Video S2.** MIZ1 movement under 0.1 *µ*M Lat B condition corresponding to Fig. 7F.

**Supplementary Video S3.** MIZ1 movement under Lat B at 1 *µ*M condition corresponding to Fig. 7K.

**Supplementary Video S4.** Effect of 10 *µ*M Lat B on MIZ1 movement, corresponding to Fig. 7P.

**Supplementary Video S5.** MIZ1 movement in *miz2* background, corresponding to Supplementary Fig. S4F.
