# Supplementary material for: Intracellular MIZU-KUSSEI1 movement and hydrotropism in Arabidopsis require F-actin organization
Source: Plant Physiol. 2025 Oct 8;199(2):kiaf495. doi: 10.1093/plphys/kiaf495 (PMC12551455; doi:10.1093/plphys/kiaf495)
Supplement: kiaf495_Supplementary_Data [file kiaf495_supplementary_data.zip › Revised_legends_for_supplemental movies.docx]

**Legends for supplemental movies.**

Supplementary video 1 MIZ1 movement under control condition corresponding to Fig. 7A. Time-lapse images acquired at 200 ms exposure for a total of 100 images were compressed at 5 frames per second (fps).

Supplementary video 2 MIZ1 movement under 0.1 µM Lat B condition corresponding to Fig. 7F. The video was created by compressing at 5 fps from 100 stack images acquired for 200 ms exposure.

Supplementary video 3 MIZ1 movement under Lat B at 1 µM condition corresponding to Fig. 7K. 100 frames were compressed at 5 fps from 100 frames.

Supplementary video 4 Effect of 10 µM Lat B on MIZ1 movement, corresponding to Fig. 7P. 5 fps compressed video was created.

Supplementary video 5 MIZ1 movement in *miz2* background, corresponding to Supplementary Fig. S4F.
