# Supplementary material for: Intracellular MIZU-KUSSEI1 movement and hydrotropism in Arabidopsis require F-actin organization
Source: Plant Physiol. 2025 Oct 8;199(2):kiaf495. doi: 10.1093/plphys/kiaf495 (PMC12551455; doi:10.1093/plphys/kiaf495)
Supplement: kiaf495_Supplementary_Data [file kiaf495_supplementary_data.zip › Supplementary_materials_Final.pdf]

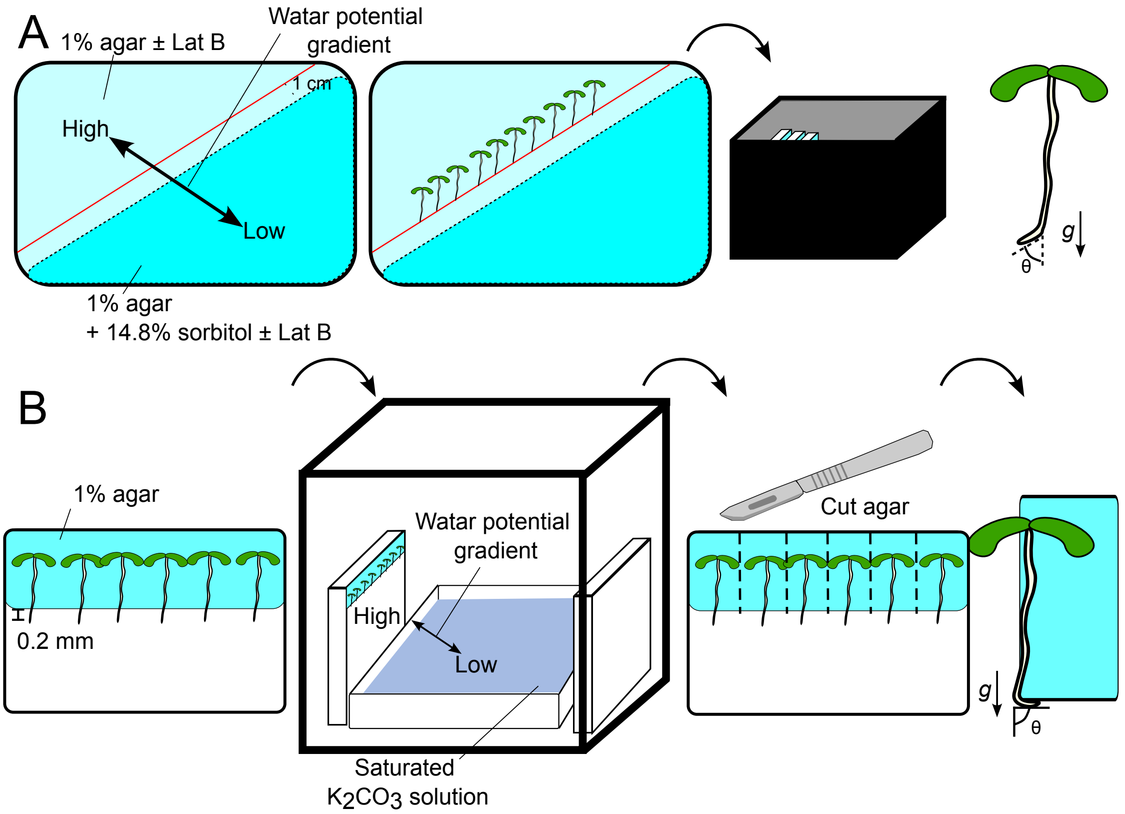

Supplementary Figure S1 Hydrotropism experimental systems.

(A) Split-agar-based hydrotropism assay. A schematic illustration of the split-agar setup is shown. A 1% (w/v) agar plate (top) and a 1% (w/v) agar plate containing sorbitol (bottom) were placed side-by-side diagonally in a rectangle dish. Seedlings were placed on the plain agar with their root tips positioned 1 cm away from the junction between the two agar blocks (indicated by the red line). The plates were sealed with surgical tape and incubated at 23°C in the dark. Root curvature was recorded every twelve hours using a flatbed scanner.

(B) Humidity-based hydrotropism assay. Seedlings were aligned on 1% (w/v) agar with their root tips suspended 0.2 mm from the edge of the agar. To create a low-humidity environment, a plastic tray containing saturated K_2CO_3 solution was placed in an acrylic chamber prior to the experiment. The plates were incubated in the chamber at 23°C in the dark. After two, four, and eight hours, the plates were removed, and the agar was cut to isolate individual seedlings. The roots were photographed under a microscope, and the root curvatures were measured from the captured images.

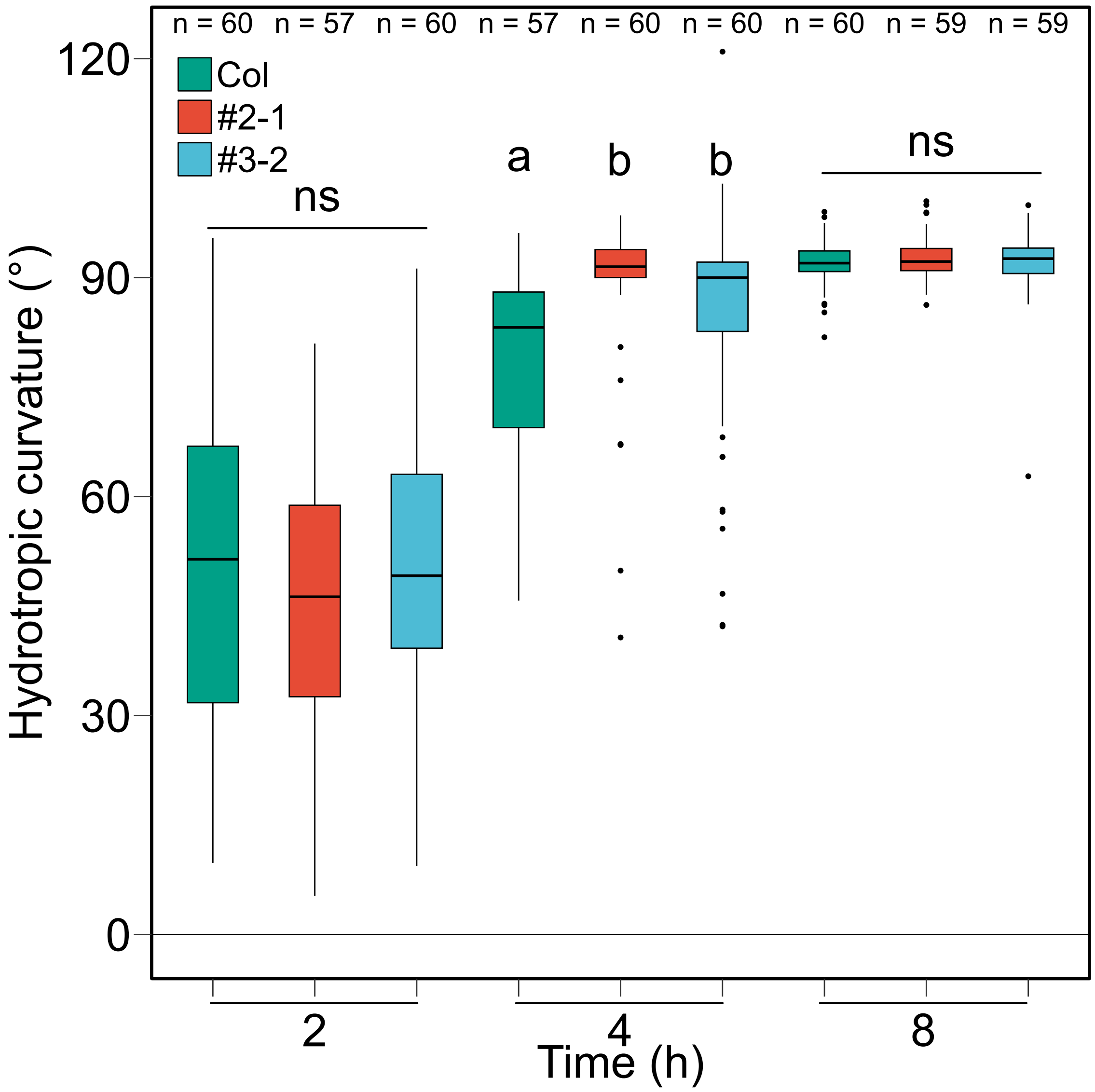

Supplementary Figure S2. Hydrotropic responses of *ADF1-4Ri* #2-1 and #3-2 in the humidity-based hydrotropism assay.

Seedlings were subjected to the humidity-based hydrotropism assay described in Supplemental Fig. S1B. The experiments were independently repeated three times using approximately 20 seedlings per experiment. The number of seedlings analyzed is shown in the graph. Boxplots represent the interquartile range (first to third quartiles); whiskers indicate the minimum and maximum values. The line within each box indicates the median, and outliers are shown as dots. Different letters denote statistically significant differences at each time point, determined by Tukey's HSD test ($p < 0.05$).

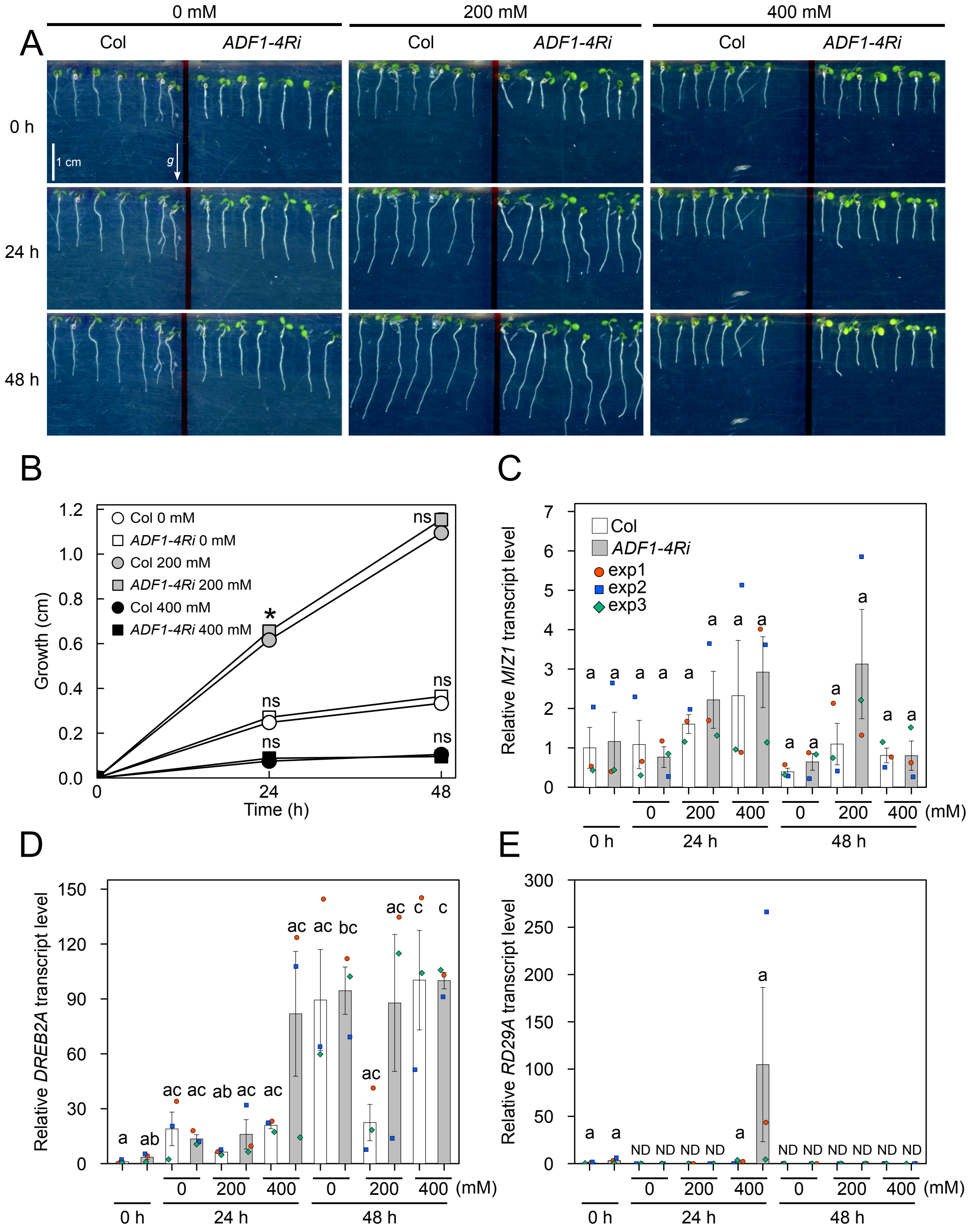

Supplementary Figure S3 Effect of osmotic stress treatment on Col and *ADF1-4Ri*#3-2.

(A) Representative images of Col and *ADF1-4Ri*#3-2 grown under various concentrations of sorbitol (0, 200, 400 mM). An arrow shows the direction of gravity. (B) Root growth of wild type and *ADF1-4Ri* grown under non-stress (0 mM) or osmotic stress (200- or 400 mM sorbitol) conditions. Data represents means ± SE across three

independent experiments (n = 74 – 75). An asterisk shows the statistically significant difference between the same treatment at the same time point (Welch's *t*-test; $p < 0.05$, ns; not significantly different $p > 0.05$). (C–E) mRNA levels of *MIZ1* and the osmotic stress responsive genes (*DREB2A* and *RD29A*) in Col and *ADF1-4Ri*#3-2 grown under various concentrations of sorbitol (0, 200, 400 mM). Data represent means ± SE, and raw data across three independent experiments are spotted in different colors. Different letters annotated in graph indicate statistically significant differences determined by Tukey's honestly difference test ($p < 0.05$). ND: not detected.

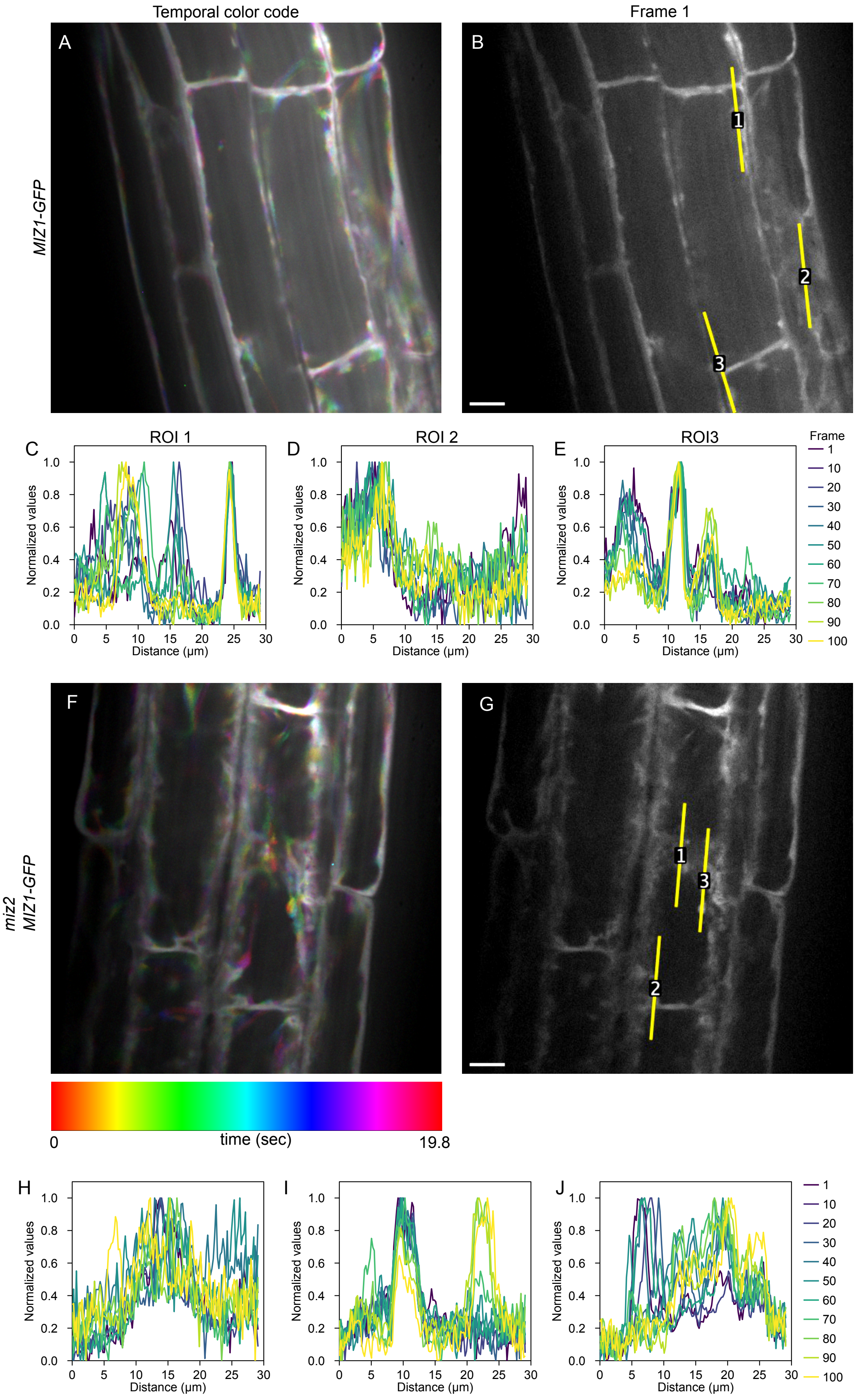
Temporal color code
Frame 1
A
B
MIZ1-GFP
1
2
3
C
D
E
ROI 1
ROI 2
ROI3
Normalized values
Distance (µm)
Frame
1
10
20
30
40
50
60
70
80
90
100
F
G
miz2
MIZ1-GFP
time (sec)
0
19.8
H
I
J

Supplementary Figure S4 Effect of *miz2* mutation on subcellular dynamics of MIZ1.

MIZ1-GFP and *MIZ1-GFP* in *miz2* (*miz2* MIZ1-GFP) were observed using confocal microscopy, and time-lapse images on the cortical cells were acquired at 200 ms intervals for a total of 100 frames. Panel A and F represent the temporal color-coded images for MIZ1 movement. Panel B and G show the images of the first frame. Yellow lines indicate the region of interests (ROIs) used for the movement analyses (C to E and H to J). Scale bar = 10 μm. Fluorescent intensity profiles along the yellow lines, drawn from the bottom to the top in (B) and (G) are shown in (C to E) and (H to J), respectively.

Supplemental Tables

Supplementary Table S1. Primer sets used for genotyping.

Name	Sequence (5’ – 3’)	Target allele
ADF1-4Ri_NI478	TGATGCGGATCAACAGGTG	*ADF1-4Ri*
ADF1-4Ri_NI479	TGATGGTATCGGTGTGAGC	*ADF1-4Ri*
MIZ1 genotype F	AACACTCATCATCACTTTGG	*miz1-1*
MIZ1 genotype R	ATCTCATGACACAACATTGC	*miz1-1*
MIZ2 genotype F	CTGGTCCAACCATTGCTGCC	*miz2*
MIZ2 genotype R1	TTGATTCCGTCTACTCCGCC	*miz2*

Supplementary Table S2. Primer set for RT-qPCR.

Name	Sequence (5’ – 3’)	Target gene
MIZ1_RT_F	TCTCTCCTCCTCATCGTCGG	*MIZ1*
MIZ1_RT_R	CAGGTCAAGGAGCAAGACCG	*MIZ1*
rRNA_RT_F	ATAAACGATGCCGACCAGGG	*rRNA*
rRNA_RT_R	TTAAGCCGCAGGCTCCACTC	*rRNA*
RD29A_RT_F	GGATTTGACGGAGAACCAGA	*RD29A*
RD29A_RT_R	GGATGAGAAAGTTCCGGTGA	*RD29A*
DREB2A_RT_F	AGACTATGGTTGGCCCAATG	*DREB2A*
DREB2A_RT_R	AGTTGATTCTTTGTAGCGGA	*DREB2A*
RAB18_RT_F	ACTGAAGGCTTTGGAACTGG	*RAB18*
RAB18_RT_R	TGACCTGGCAACTTCTCCTT	*RAB18*
